# High concentrate diets altered the structure and function of rumen microbiome in goats

**DOI:** 10.3389/fmicb.2024.1416883

**Published:** 2024-07-31

**Authors:** Jinju Mao, Lizhi Wang, Zhisheng Wang, Bai Xue, Quanhui Peng, Rui Hu, Jianxin Xiao

**Affiliations:** Animal Nutrition Institute, Sichuan Agricultural University, Chengdu, China

**Keywords:** high concentrate diets, rumen, rumen fermentation, rumen microbiome, microbial function

## Abstract

This study used metatranscriptomics to investigate the effects of concentrate diet level on rumen microbiome composition and function in goats. A total of 12 healthy 120-day-old Da’er goats were randomly allotted into two treatments: L group (low dietary concentrate level group, concentrate: forage ratio was 25: 75) and H group (high dietary concentrate level group, concentrate: forage ratio was 80: 20). The study included a 10-day pre-feeding period and a 60-day growth experiment. The results showed that compared with the L group, the average daily gain and the slaughter rate in the H group were increased, while the F/G was decreased; the concentration of lactate and ammonia nitrogen, and the proportion of butyrate and valerate in the rumen of the H group were increased, while the proportion of acetate, and the ratio of acetate to propionate were decreased (*p* < 0.05). Among rumen bacteria, compared with the L group, the H group significantly decreased the relative abundance of Firmicutes and Fibrobacteria at the phylum level, decreased the relative abundance of *Bacteroidetes*, *Fibrobacter*, and *Sarcina* and increased the relative abundance of *Clostridium* at the genus level, and decreased the relative abundance of *Fibrobacter succinogenes*, *Sarcina* sp. DSM 11001, *Oscillibacter* sp. KLE 1728, and *Ruminococcus flavefaciens* and increased the relative abundance of Clostridium sp. ND2 and Firmicutes bacteria CAG: 103 at the species level (*p* < 0.05). Among rumen fungi, the relative abundance of Basidiomycota, Neocallimastigomycota, *Mortierella*, *Mortierella* elongata, and Gonapodyna prolifera was lower in the H group than that in the L group (*p* < 0.05). Functional annotation results showed that the abundance of Glycoside hydrolases genes in rumen microbiome was significantly decreased in the H group compared to the L group (*p* < 0.05). The result of KEGG DEGs enrichment analysis showed that the gene expression of cellulose 1,4-β-cellobiosidase, acetyl-CoA hydrolase, lactate dehydrogenase, succinate-semialdehyde dehydrogenase, D-malate dehydrogenase and related genes in methane production pathways of rumen microbiome was decreased in the H group. In summary, feeding high concentrate diets improved the production performance of goats, altered the structure and composition of rumen microbiome and changed the function of rumen microbiome.

## 1 Introduction

In China, it is common for farmers to feed large amounts of concentrate feed to cattle and sheep in order to meet the demand for nutrients, improve their production performance and maximise economic benefits. Especially during the fattening period for meat goats, the proportion of concentrate feed in the diet is even higher than 80% (Tian et al., 2019; Shah et al., 2022). Numerous studies have shown that feeding high concentrate diet (HCD) results in a change in rumen fermentation type from acetate to propionate (Zhang X. et al., 2020; Jiang et al., 2021) and a decrease in methane production (Bannink et al., 2006; Beauchemin et al., 2008). However, there are no relevant studies that provide an in-depth explanation of these phenomenons. This is most likely due to the fact that HCD causes alterations in the composition and structure of rumen microbial composition and structure, leading to dysfunction (Liu et al., 2015; Zhang R. et al., 2020; Pang et al., 2022; Wang et al., 2023). It is well known that the large microbial community in the rumen of ruminant animals not only aids in digestion and absorption of nutrients from the feed (Henderson et al., 2015; Liu et al., 2017; Shi et al., 2023), but also maintains the host’s health by balancing and stabilizing the rumen microbial community (Jami and Mizrahi, 2012). This is also confirmed by studies on goats (Zhang et al., 2017; Luo et al., 2022).

Currently, although there have been numerous researches on the effect of dietary concentrate to forage ratio on rumen microbiome (Fernando et al., 2010; Wang L. et al., 2020), most of these studies have used 16 s rRNA sequencing technology, which can only provide information about the effects of diets on the structure and composition of rumen bacteria, but cannot adequately reveal the effects of diets on the functions of rumen microbiome. Furthermore, some researchers have also studied the effects of HCD on rumen microbial composition and function in ruminants based on macrogenomics, but this technique cannot distinguish between microorganisms that is actively growing and metabolising and those that are dormant or dead, thus failing to depict the real-time microbial status (Singer et al., 2017). However, the metatranscriptomics technique can overcome the above shortcomings. Therefore, in this experiment, goats were used as the experimental animals, and metatranscriptomics was used to study the changes in the structural composition and function of rumen microbiome under HCD feeding conditions. The research results can provide a reference for the practical application of HCD in production.

## 2 Materials and methods

The ethics of the experimental were approved by the Animal Policy and Welfare Committee of the Agricultural Research Organization of Sichuan Province, China and conducted in accordance with the guidelines of the Animal Care and Ethical Committee of Sichuan Agricultural University. ARRIVE guidelines and National guidelines and regulations (GB 14925-2010) were followed for all animal experiments.

### 2.1 Animal, diet and experiment design

This trial was performed at the Animal Nutrition Institute, Sichuan Agricultural University. A total of 12 healthy 120-day-old Jianzhou Da’er goats (6 males, 6 females) with an initial body weight (BW) of 20.9 ± 1.5 kg was used as the experimental animals. A single factor treatment design was adopted, according to the different ratio of concentrate to forage, animals were divided into two groups: low dietary concentrate level group (L group) fed with a diet contains 25% concentrate and high dietary concentrate level group (H group) fed with a diet contains 80% concentrate. The study included a 10-day pre-feeding period and a 60-day growth experiment. During the whole experiment, the goats were reared in single-column and had free access to feed and water.

The diet was formulated based on the feeding standard of Meat-Producing Sheep and Goats of Chinese agricultural industry standards (NY/T 816-2004). The dietary energy-nitrogen ratio and calcium-phosphorus ratio of the two groups was consistent. Diet composition and the nutrient level were showed in the Table 1. The nutrient levels including dry matter (DM), crude protein (CP), neutral detergent fibre (NDF), acid detergent fibre (ADF), and ether extract (EE) were measured according to GB/T6435-2014, GB/T6432-2018, GB/T20806-2006, NY/T1459-2007, and GB/T6434-2006, respectively. The contents of calcium, and phosphorus were determined according to GB/T6436-2018 and GB/T6437-2018.

**Table 1:** Composition and nutrient levels of experimental diets (basis on DM).

Items	L group	H group
**Ingredients**		
Corn	4.00	48.25
Wheat bran	7.00	7.30
Syobean meal	9.00	10.50
Rapeseed meal	1.00	1.00
Cottonseed meal	1.50	8.00
Oat grass	43.80	5.00
Wheat Straw	8.00	2.00
Alfalfa hay	23.00	14.00
CaCO_3_	0.15	1.00
CaHPO_4_	0.05	0.45
NaCl	0.50	0.50
NaHCO3	1.00	1.00
Premix^1^	1.00	1.00
Total	100.00	100.00
**Nutrient levels** ^2^		
DM/%	90.14	87.84
CP/%	12.06	15.93
EE/%	5.23	3.08
CF/%	23.19	8.71
NFE/%	35.24	50.89
DE/(MCal.kg^−1^ DM)	2.17	2.88
NDF/%	43.54	19.55
ADF/%	21.05	7.34
Ca/%	0.59	0.76
P/%	0.37	0.49

1 The premix provided the following per kg of diet: vitamin A 2200 IU; Vitamin D 250 IU; Vitamin E 20 IU; Fe 40 mg; Cu 10 mg; Zn 30 mg; Mn 40 mg; I 0.8 mg; Se 0.2 mg; Co 0.11 mg.

2 DE was calculated value, while the others were measured values.

### 2.2 Production performance

At the beginning of the growth experiment, the initial body weight (IBW) was recorded on an empty stomach. During the growth experiment, the feed intake and remaining feed were recorded every 10 days, and then the average daily feed intake (ADFI) was calculated. Accordingly, the body weight (BW) was weighed and recorded after fasting for 12 h every 10 days, the average daily gain (ADG) was calculated, and then the F/G (feed intake/gain) was calculated.

All goats were slaughtered on the second day after the digestive experiment. During the slaughter process, feeding was carried out based on the sampling time of 2 h after morning feeding. After removing the head, hooves, blood, fur, and internal organs, the carcass weight was weighed and recorded, and then the slaughter rate was calculated.

Slaughterrate=carcassweight/pre−slaughterliveweight×100%.

### 2.3 Sample collection

After slaughter, the rumen was removed. A part of ruminal contents was squeezed into 2 sterile tubes, frozen in liquid nitrogen, and stored at −80°C until further microbiome analysis. The ruminal contents were collected using a sterile beaker, and then the pH was quickly determined. The ruminal fluid was strained through 4 layers of gauze, divided into 4 tubes of 50 mL centrifuge tubes, and stored in a refrigerator at −20°C for ruminal fermentation parameter measurements.

### 2.4 Ruminal fermentation parameters

The pH value of ruminal fluid was immediately determined by thundermagnetic pH meter (PHBJ-260, China). The volatile fatty acids (VFAs) content, including acetate, propionate, butyrate, and valerate, in ruminal fluid were measured by gas chromatograph (GC-2014FRGA1, Shimadzu, Tokyo, Japan). The ammonia nitrogen (NH_3_-N) concentration in ruminal fluid was determined by colorimetric technique (Broderick and Kang, 1980). The lactate (LA) content of ruminal fluid was measured using commercial ELISA kits (Jiangsu Meimian Biotechnology Co., Ltd), and all assay procedures were performed according to the instructions.

### 2.5 Metatranscriptome analysis of ruminal microorganisms

#### 2.5.1 RNA extraction and metatranscriptomic sequencing

The microbial total RNA was extracted from ruminal content samples using the TRIzol reagent (Invitrogen Life Technologies, USA) as described by Han-Song (Yu et al., 2005). RNA integrity was analyzed by agarose gel electrophoresis to avoid DNA contamination and RNA purity was detected by NanoPhotometer spectrophotometer. Then the RNA was quantified using Qubit V2.0 Fluorometer, while the RIN (RNA integrity number) was determined using the Agilent 2,100 Bioanalyzer (Agilent, USA).

The RNA samples were initially treated to deplete the rRNA and enrich mRNA. Subsequently, the cDNA library was established after RNA fragmentation, cDNA first-strand synthesis, cDNA second-strand synthesis, cDNA purification, end repair, adenylation, and PCR enrichment. Preliminary quantification, dilution (1.5 ng/μl), insert size detection, and accurate quantification were carried out to ensure the quality of the cDNA library. Then, the metatranscriptomic library was sequenced using an Illumina HiSeq 4,000 platform (paired-end; read length, 150 bp).

#### 2.5.2 Metatranscriptome data analysis

The raw data were subjected to filtering of host reads, adapter sequences or poly-N and low-quality (Qpherd ≤20) sequences. The filtered Clean Reads were carried out strict quality control to ensure that the sequencing error rate of a single base position is less than 1%, with a maximum of 6%. The clean reads obtained from pre-processing were compared against the NCBI rRNA, tRNA and SILVA databases to isolate rRNA sequences. The remaining mRNA sequence reads were assembled *de novo* into transcripts using Trinity, combined and clustered into unique classes with CD-HIT-EST at 95% identity, and then the unigene collection was obtained (He et al., 2019).

#### 2.5.3 Taxonomic annotation

The unigenes was performed against the bacteria and fungi of the NCBI NR (Version: 2016-11-05) database (blastp, evalue ≤ *e*^−5^) using DIAMOND software (Buchfink et al., 2015). The total gene abundance of corresponding to the species was obtained, and then calculated the relative abundance of different species in each sample at different taxonomic levels. Bacterial and fungal compositional profiles were summarised at the phylum, genus and species levels. And the alpha and beta diversity indexes were calculated by R (Version 4.1.2, https://www.r-project.org).

#### 2.5.4 Functional annotation

In order to study the adaptability of rumen microbiome in goats to diets with different concentrate levels, the unigenes were annotated using DIAMOND by aligning with the CAZymes (Carbohydrate-Active Enzymes), GO (Gene Ontology), and KEGG (Kyoto Encyclopedia of Genes and Genomes) database (blastp, evalue ≤ *e*^−5^). CAZymes were annotated using hmmscan^1^ against the CAZymes database, including Glycoside hydrolases (GHs), Glycosyl transferases (GTs), Polysaccharide lyases (PLs), Carbohydrate esterases (CEs), Auxiliary activities (AAs), and Carbohydrate-binding modules (CBMs). GO were annotated using blast2go,^2^ including Molecular function (MF), Cellular component (CC), and Biological process (BP). For the KEGG function classification, unigenes were annotated into five categories of KEGG metabolic pathways, including cellular processes, environmental information processing, organismal systems, metabolism and genetic.information processing.

#### 2.5.5 Differential expressed genes (DEGs) analysis

The Clean reads of each sample were mapped with the reference sequence using root-mean-square error (RSEM) to further obtain the readcount number of each sample aligned to each gene. Fragments Per Kilobase of transcript per Million mapped reads (FPKM) were performed to analyze the gene expression level (Wang H. et al., 2020). Then, the readcount was conducted normalization, calculated the hypothesis test probability (*p*-value), and carried out multiple hypothesis test correction to obtain the FDR using the DEseq. *p*-value was calculated according to the calculation model of negative binomial distribution, and the corrected *p*-value (*P*adj) < 0.05 was used as the differential gene screening standard.

Finally, the GO enrichment analysis was performed using Goseq and the KEGG pathways enrichment analysis was conducted using KOBAS to research the related biological functions or pathways. The *p*-value was calculated according to the hypergeometric distribution formula as follows:

$$P=1-\overset{m-1}{\underset{i=0}{\sum }}\frac{\left(\begin{array}{c} M\\ i \end{array}\right)\left(\begin{array}{c} N-M\\ n-i \end{array}\right)}{\left(\begin{array}{c} N\\ n \end{array}\right)}$$

*i* = Gene of a pathway; *n* = Differential Gene-set; *N* = Background Gene-set; *M* = Gene of a pathway.

#### 2.5.6 Validation of expression of selected DGEs using qRT-PCR

Samples were selected from the same batch of experiments for RNA extraction and reverse transcription by qRT-PCR as described by Wang et al. (2017). The primer sequence was synthesized by Shenggong Biotechnology Co., Ltd. (Shanghai) based on the NCBI database, as detailed in Supplementary Table 1. PCR reaction was performed using a three-step method, with a reaction procedure of 95°C, 10 min, and 1 cycle; 95°C, 15 s, 40 cycles; 60°C, 60 s, 40 cycles. β-Actin was used as an internal reference gene using 2⁻^ΔCT^ to calculate the relative expression level of the target gene. The qRT-PCR validation results showed a high degree of consistency with the results of transcriptome sequencing.

### 2.6 Statistical analysis

All experiment data were collated and preliminary processed using Microsoft office 2019. Growth performance, slaughter rate and ruminal fermentation parameters were shown as Mean ± SD, and the relative abundance of ruminal microorganisms was shown as Mean ± SEM. The differences in the growth performance, slaughter rate, and ruminal fermentation parameters between groups were analyzed using two sample unpaired T-tests and the differences in microbial relative abundance and functional gene relative abundance between groups were analyzed using Mann–whitney u test in SPSS 25.0. *p* < 0.01 indicates highly significant differences, 0.01 ≤ *p* < 0.05 indicates significant differences, and *p* ≥ 0.05 indicates insignificant differences.

## 3 Results

### 3.1 Production performance and ruminal fermentation parameters

The results of production performance and fermentation parameters are shown in Table 2. There was no significant difference in the final body weight and the ADFI of goats (*p* > 0.05). The ADG of the H group was significantly higher than that of the L group (*p* = 0.023). The F/G was lower and the slaughter rate was higher in goats fed HCD compared to goats fed LD (*p* < 0.01). Compared with the L group, the concentration of LA and NH_3_-N and the proportion of butyrate and valerate in the H group significantly increased (*p* < 0.001), while the proportion of acetate (*p* = 0.001) and the ratio of acetate to propionate (*p* = 0.035) significantly decreased.

**Table 2:** Effects of concentrate diet levels on production performance and rumen fermentation parameters of goats (*n* = 6).

Item	L group	H group	*p*-value
Initial body weight (kg)	20.32 ± 2.22	21.53 ± 2.71	0.415
Final body weight (kg)	28.68 ± 1.93	31.7 ± 3.98	0.125
Average daily gain (g/d)	139.44 ± 11.04	169.44 ± 25.07	0.015
Average daily feed intake (g/d)	1172.79 ± 130.78	1069.50 ± 172.33	0.267
Feed intake/gain (%)	8.46 ± 1.19	6.31 ± 0.31	0.002
Slaughter rate (%)	0.49 ± 0.03	0.56 ± 0.03	0.001
pH	6.13 ± 0.14	5.97 ± 0.16	0.094
Lactic Acid (mg/L)	13.70 ± 9.66	55.27 ± 32.77	<0.001
NH_3_-N (mg/100 mL)	14.96 ± 2.00	42.48 ± 7.67	<0.001
Total SCFA (mmol/L)	116.18 ± 12.09	119.29 ± 9.76	0.635
Acetate %	65.00 ± 2.26	59.43 ± 2.15	0.001
Propionate %	19.59 ± 1.40	21.22 ± 2.43	0.211
Isobutyrate %	0.83 ± 0.23	0.84 ± 0.10	0.925
Butyrate %	12.81 ± 1.70	19.01 ± 2.66	<0.001
Isovalerate %	0.92 ± 0.27	1.12 ± 0.15	0.149
Valerate %	0.86 ± 0.06	1.12 ± 0.09	<0.001
Acetate: Propionate	3.34 ± 0.34	2.77 ± 0.36	0.035

L group = low concentrate group; H group = high concentrate group. *p* < 0.01 indicates highly significant differences, 0.01 ≤ *p* < 0.05 indicates significant differences, and *p* ≥ 0.05 indicates insignificant differences.

### 3.2 Rumen microbiome structure and composition

#### 3.2.1 Analysis of rumen microbiome diversity

**Figure 1:**
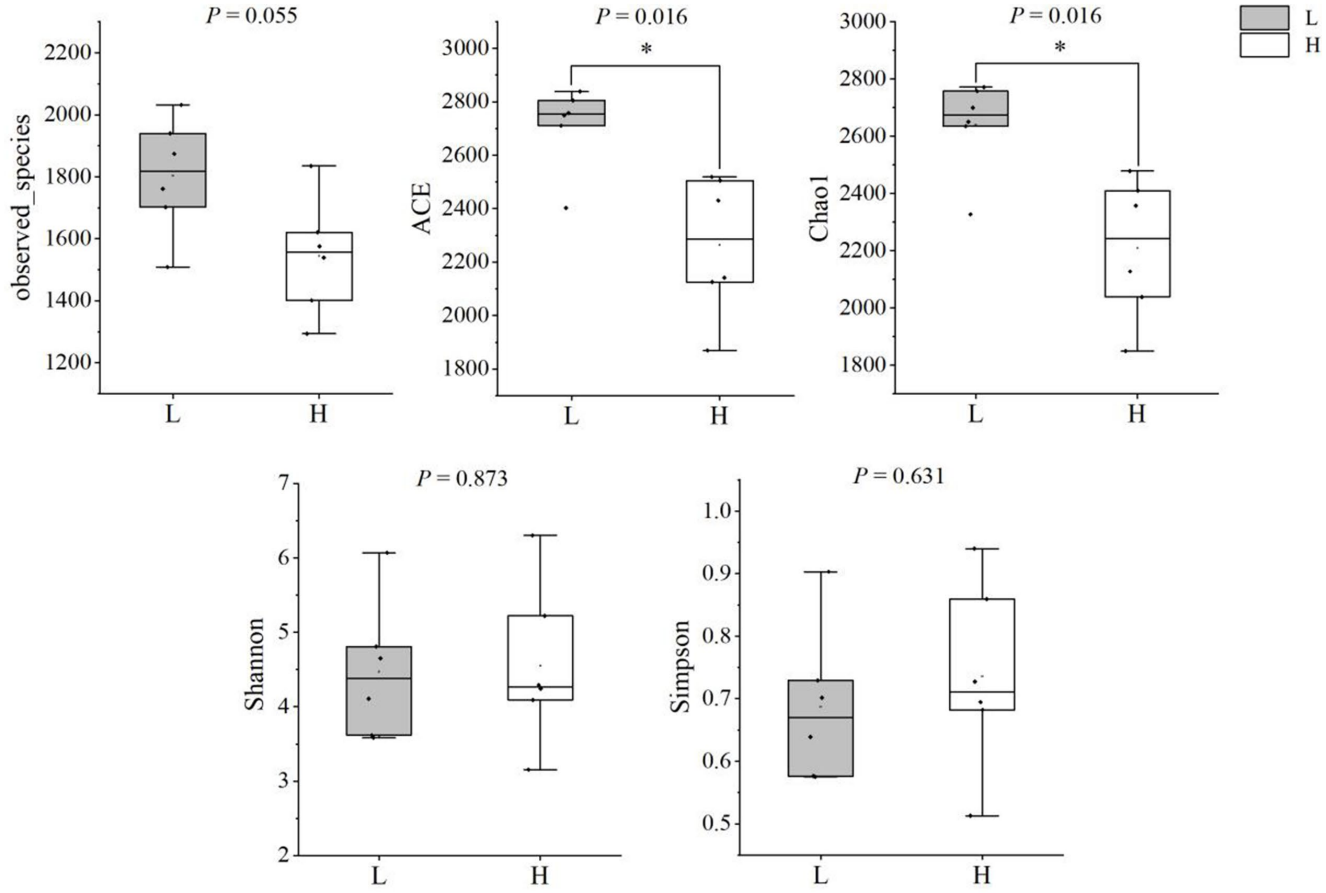
Comparison of alpha diversity indices between groups. L group = low concentrate group; H group = high concentrate group. *Significant (0.01 ≤ *p* < 0.05).

**Figure 2:**
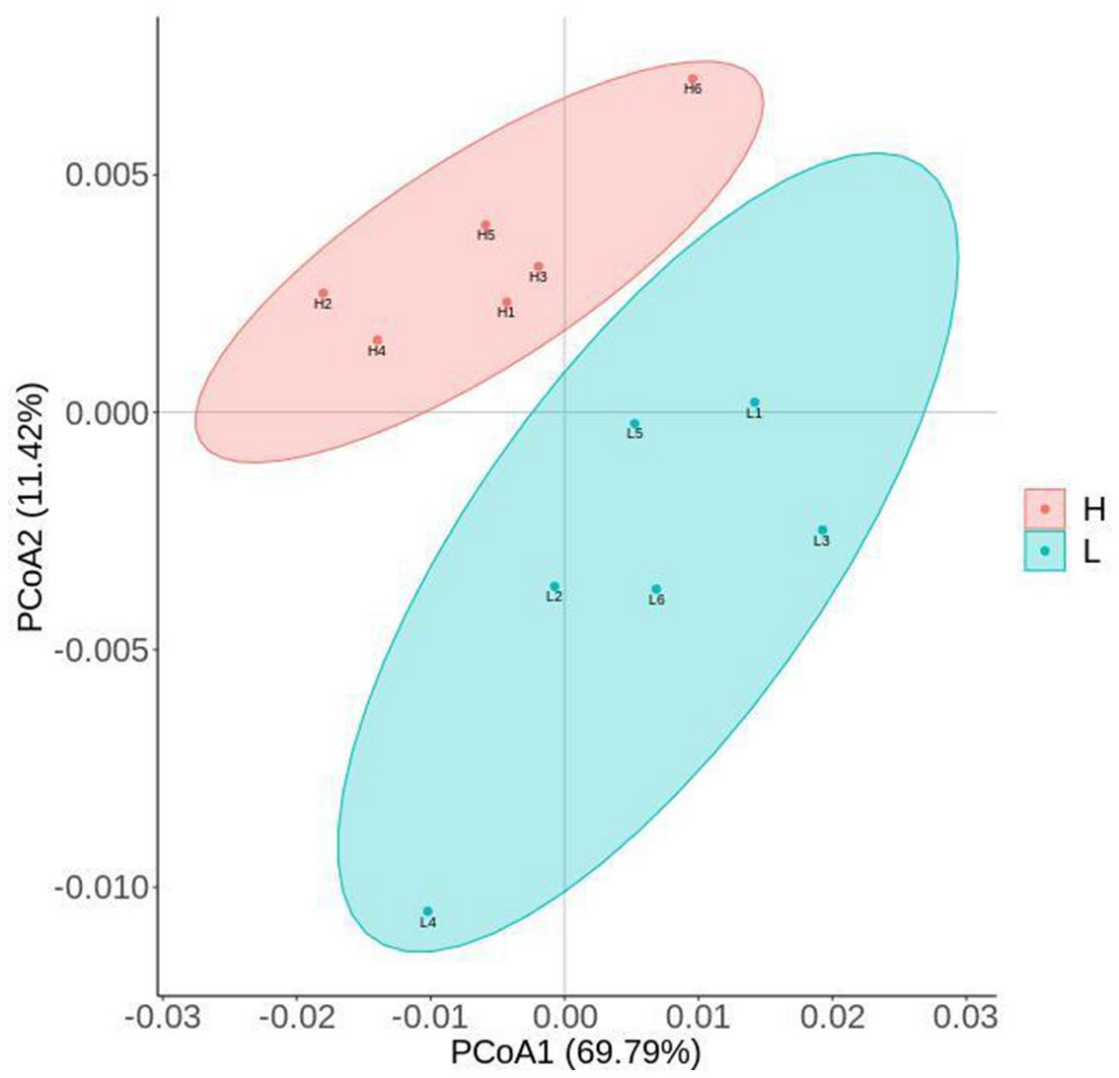
PCoA (Principal coordinate analysis) cluster analysis of rumen microbiome. L group = low concentrate group; H group = high concentrate group.

The results of alpha-diversity indexes in rumen microbiome are shown in Figure 1, including observed_species, ACE, Chao1, Shannon, and Simpson. The results indicated that the ACE and Chao1 indexes in the L group were higher than that in the H group (*p* = 0.016), which suggested the microbial richness in the L group was higher than that in the H group. As shown in Figure 2, the Principal coordinate analysis (PCoA) showed that the samples of the L group and H group were in distinct clusters, indicating the structure of rumen microbiome was different between the L group and H group based on the Bray-Curtis.

#### 3.2.2 Differences of rumen microbial composition at kingdom level between groups

**Figure 3:**
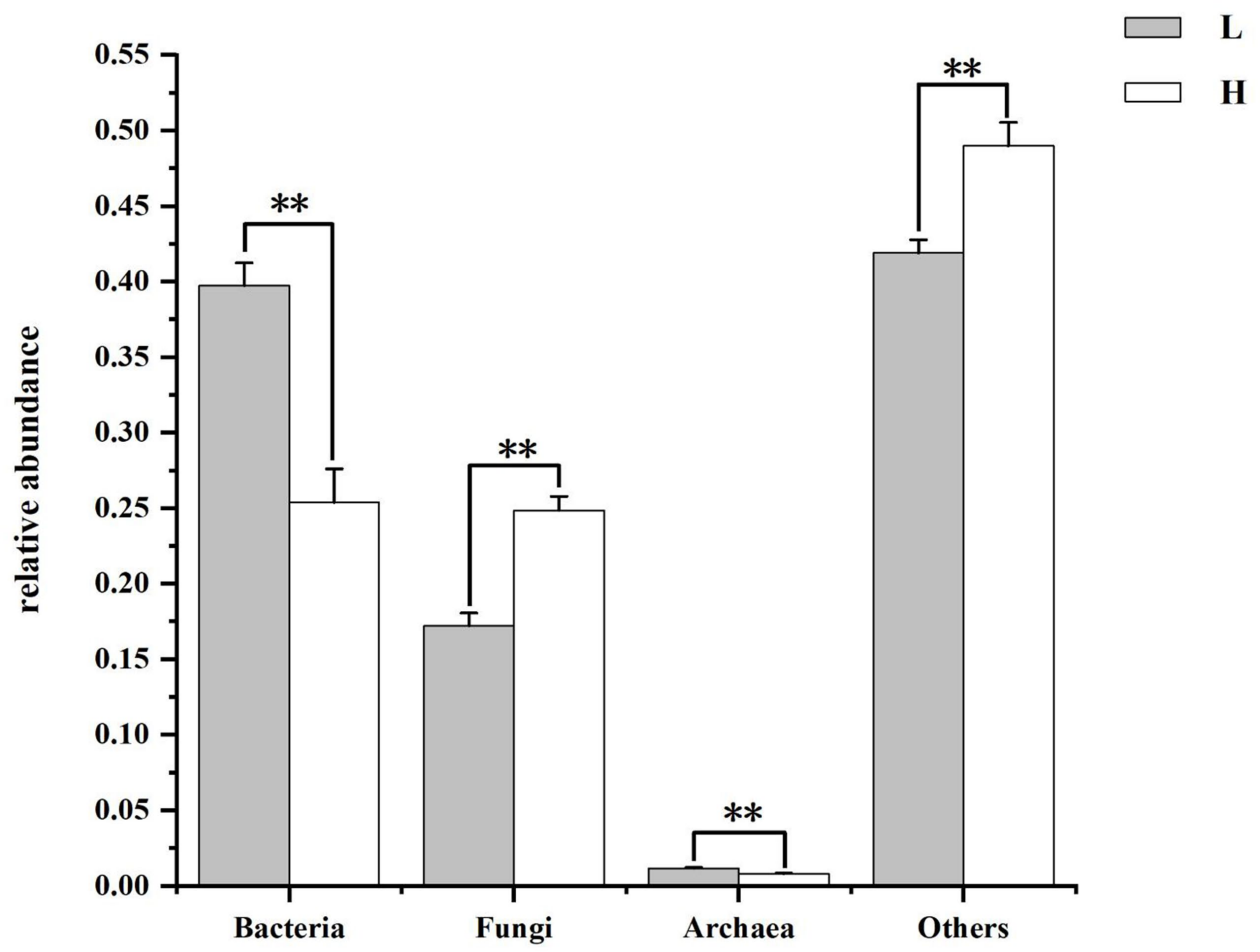
Composition and differences of rumen microbiome between groups at kingdom level. L = low concentrate group; H = high concentrate group. **Highly significant (*p* < 0.01); *Significant (0.01 ≤ *p* < 0.05).

Figure 3 shows composition and differences of rumen microbiome at kingdom level. Bacteria and fungi were the dominant microorganisms in the rumen of groups. The relative abundance of bacteria and archaea was lower and the relative abundance of fungi and others was higher in goats fed HCD compared to goats fed LD (*p* < 0.01).

#### 3.2.3 Differences of rumen bacterial composition between groups

**Figure 4:**
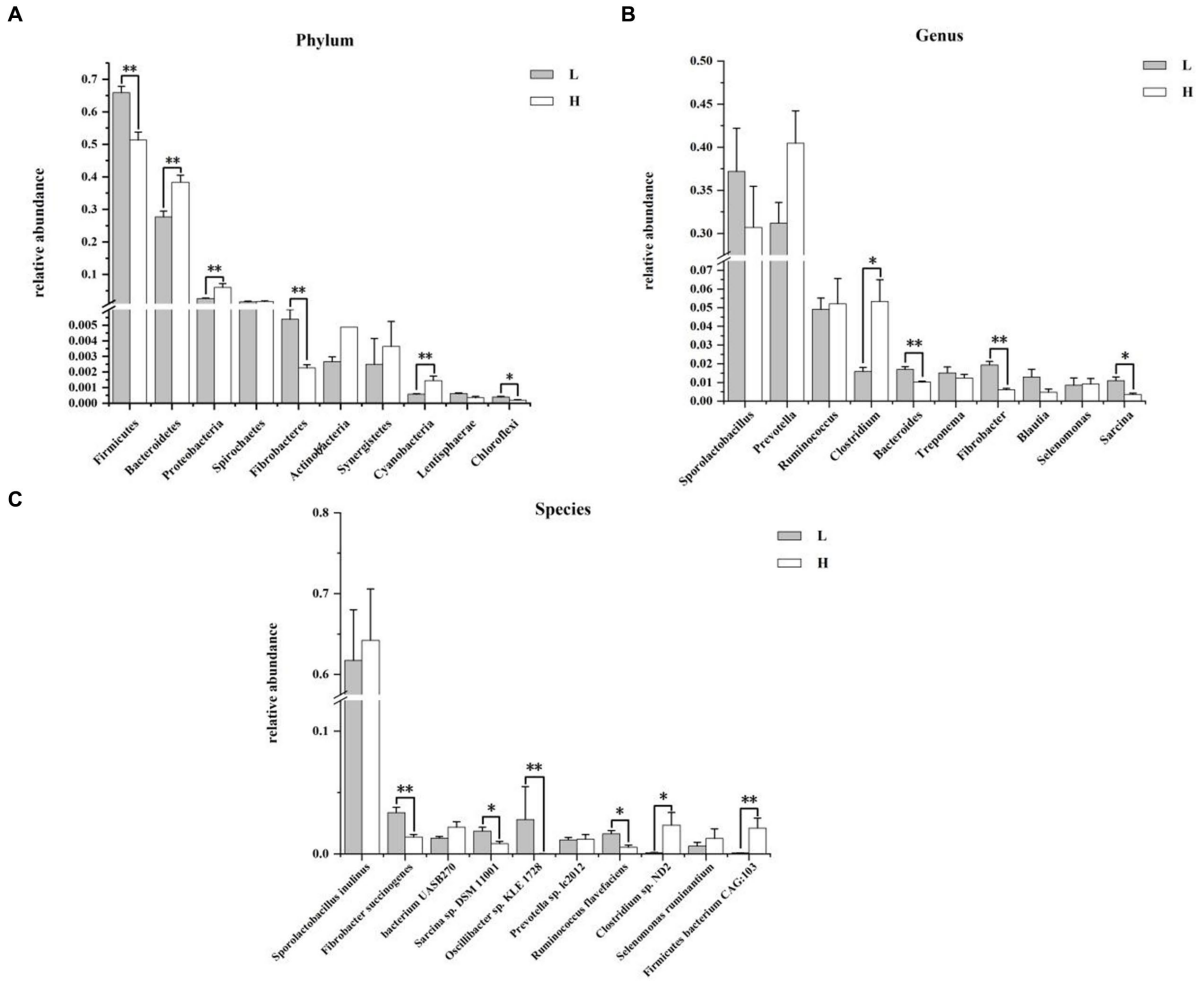
Composition and differences of rumen bacteria between groups. **(A)** Composition and differences of dominant rumen bacteria (top 10) between groups at phylum level; **(B)** Composition and differences of dominant rumen bacteria (top 10) between groups at genus level; **(C)** Composition and differences of dominant rumen bacteria (top 10) between groups at species level. L = low concentrate group; H = high concentrate group. **Highly significant (*p* < 0.01); * Significant (0.01 ≤ *p* < 0.05). Only the top 10 of the relative abundance of rumen bacteria are shown in the figure.

Figure 4 shows the composition and differences of rumen bacteria between groups. At the phylum level (Figure 4A), the dominant bacteria were Firmicutes, Bacteroidetes, Proteobacteria, and Spirochaetes in order between the two groups. Compared with the L group, the relative abundance of Firmicutes (*p* = 0.006), Fibrobacteres (*p* = 0.004), and Chloroflexi (*p* = 0.014) of the H group were significantly lower, and the relative abundance of Bacteroidetes, Proteobacteria, and Cyanobacteria of the H group were significantly higher (*p* < 0.010). Figure 4B showed the genus level of rumen bacteria (top 10), *Sporolactobacillus* and *Prevotella* were the major bacteria in rumen. Compare to the L group, the H group showed a significantly higher relative abundance of *Clostridium* (*p* = 0.037), while the relative abundance of *Bacteroides* (*p* = 0.004), *Fibrobacter* (*p* = 0.004), and *Sarcina* (*p* = 0.016) was significantly lower, and the relative abundance of *Prevotella* washigh in the H group by 22.91% compared to the L group (*p* = 0.078). At the species level (Figure 4C), the most predominant species was *Sporolactobacillus inulinus* in the rumen. The relative abundance of *Fibrobacter succinogenes* (*p* = 0.006), *Sarcina* sp. *DSM 11001* (*p* = 0.037), *Oscillibacter* sp. *KLE* 1728 (*p* = 0.003), and *Ruminococcus flavefaciens* (*p* = 0.010) in H group was lower than that in L group. In addition, the relative abundance of *Clostridium* sp. *ND2* (*p* = 0.016) and *Firmicutes bacterium* CAG:103 (*p* = 0.006) in H group was higher than that in L group.

#### 3.2.4 Differences of rumen fungi composition between groups

**Figure 5:**
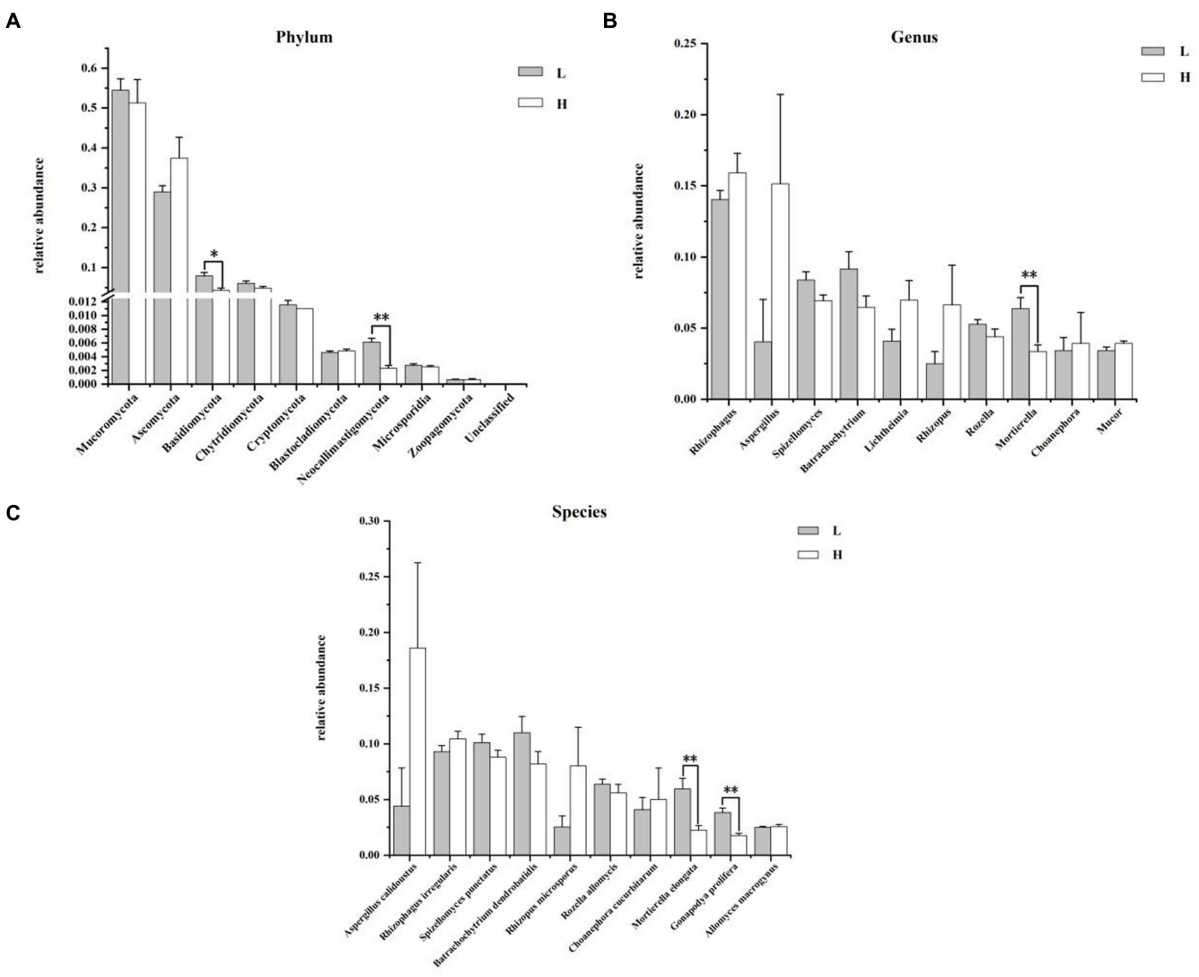
Composition and differences of rumen fungi between groups. **(A)** Composition and differences of dominant rumen fungi (top 10) between groups at phylum level; **(B)** Composition and differences of dominant rumen fungi (top 10) between groups at genus level; **(C)** Composition and differences of dominant rumen fungi (top 10) between groups at species level. L = low concentrate group; H = high concentrate group. **Highly significant (*p* < 0.01); * Significant (0.01 ≤ *p* < 0.05). Only the top 10 of the relative abundance of rumen fungi are shown in the figure.

**Figure 6:**
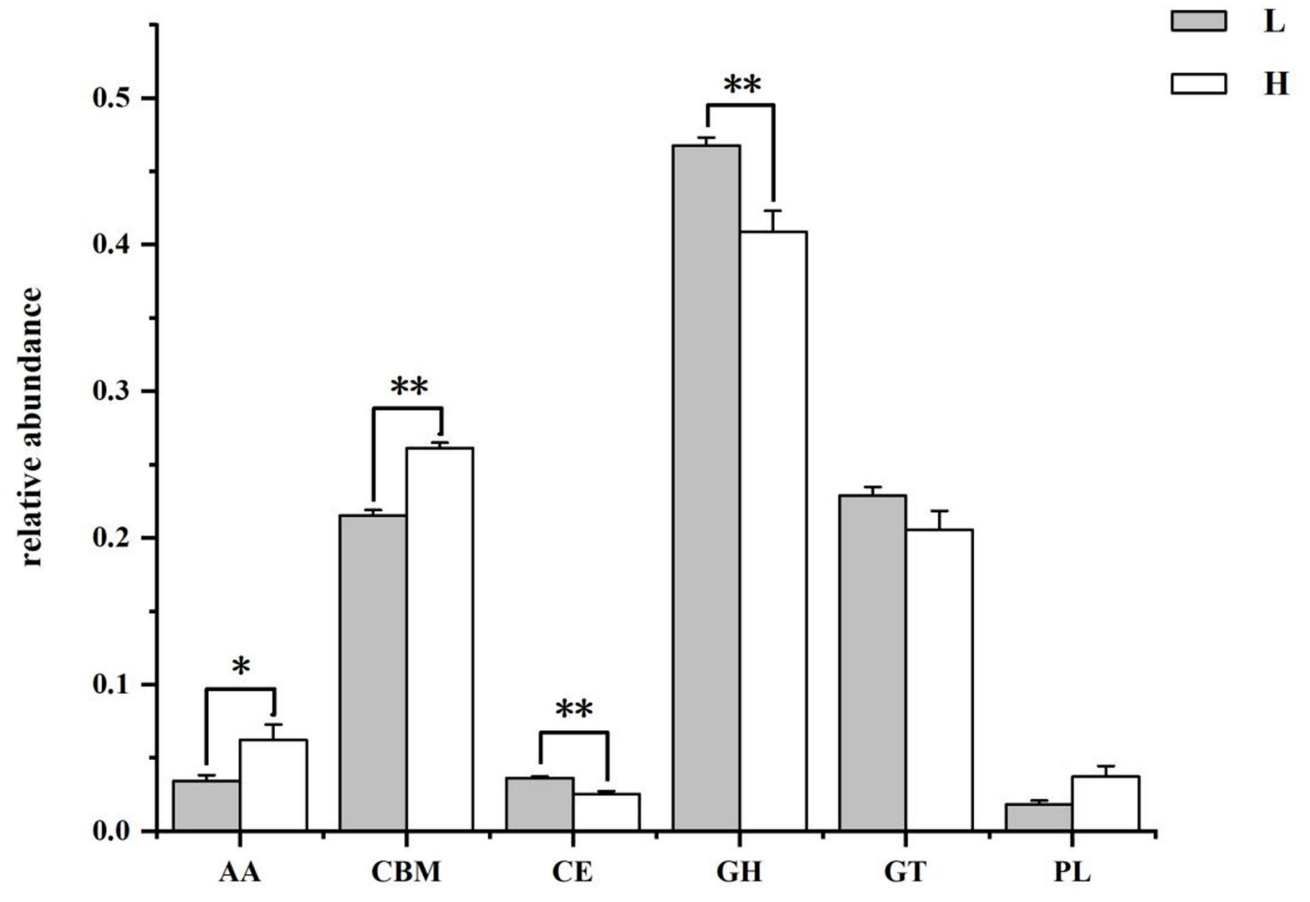
The class level annotation results of rumen microbiome genes with CAZymes database. L group = low concentrate group; H group = high concentrate group. **Highly significant (*p* < 0.01); *Significant (0.01 ≤ *p* < 0.05). AA = Auxiliary activities; CBM = Carbohydrate-binding modules; CE = Carbohydrate esterases; GH = Glycoside hydrolases; GT = Glycosyltransferase; PL = Polysaccharide lyases.

The composition and differences of rumen fungi between groups at the phylum, genus, and species levels are shown in Figure 5. At phylum level (Figure 5A), the dominant fungi were Mucoromycota and Ascomycota in the rumen. The relative abundance of Basidiomycota (*p* = 0.010) and Neocallimastigomycota (*p* = 0.004) in the H group was significantly higher than that in L group. Figure 6B illustrated that the most predominant genus fungi is *Rhizophagus*. Compared to the L group, the relative abundance of *Mortierella* in the H group was lower (*p* = 0.006). At the species level of fungi in rumen (Figure 5C), *Aspergillus calidoustus* (18.60 ± 7.66%) was the most predominant fungi in the H group, while *Batrachochytrium dendrobatidis* (11.00 ± 1.45%) was the most predominant fungi in the L group. In addition, the relative abundance of *Mortierella elongata* and *Gonapodya prolifera* in rumen of the H group was significantly lower than that of the L group (*p* < 0.01).

### 3.3 Rumen microbiome function

#### 3.3.1 CAZymes database functional annotation analysis

The results of the CAZymes gene encoding carbohydrate catabolism enzymes in the goat rumen microbiome are shown in Figure 6 and Table 3. At the class level (Figure 6), the relative abundance of CEs and GHs in the H group was lower than that in the L group (*p* = 0.004), and the relative abundance of AAs (*p* = 0.037) and CBMs (*p* = 0.004) in the H group was higher than that in the L group. The family level functional annotation result (top 20) showed that the abundance of CBM50 (*p* = 0.006), AA10 (*p* = 0.037), GH17 (*p =* 0.004), CBM43 (*p =* 0.004), GH73(*p =* 0.004), and GH23 (*p* = 0.006) in the H group was higher than that in the L group, while the abundance of GH94 (*p* = 0.006), GH32 (*p* = 0.037), GH43 (*p* = 0.004), GH13 (*p* = 0.010), GH3 (*p* = 0.004), and CBM48 (*p* = 0.006) in the H group was lower than that in the L group.

**Table 3:** The family level annotation results of CAZymes database of rumen microbiome genes.

Item	L group	H group	*p*-value
GT1	10.93 ± 0.48	11.34 ± 0.58	0.522
CBM50	4.83 ± 0.27	8.18 ± 0.63	0.006
AA10	3.29 ± 0.40	6.02 ± 1.05	0.037
GT35	3.39 ± 0.20	3.11 ± 0.34	0.262
GH17	2.20 ± 0.14	4.23 ± 0.36	0.004
CBM43	2.20 ± 0.14	4.23 ± 0.36	0.004
CBM37	2.70 ± 0.14	3.52 ± 0.36	0.078
GH5	3.03 ± 0.09	3.17 ± 0.42	0.423
GH94	3.72 ± 0.22	1.99 ± 0.31	0.006
CBM20	2.53 ± 0.20	3.09 ± 0.31	0.078
GH45	1.96 ± 0.32	3.25 ± 0.70	0.200
CBM13	2.41 ± 0.14	2.08 ± 0.16	0.078
GH9	2.09 ± 0.15	2.39 ± 0.38	0.262
GH32	3.02 ± 0.99	1.12 ± 0.19	0.037
GH43	2.56 ± 0.12	1.52 ± 0.07	0.004
GH73	0.89 ± 0.06	3.11 ± 0.29	0.004
GH13	2.64 ± 0.27	1.26 ± 0.21	0.010
GH3	2.47 ± 0.09	1.21 ± 0.08	0.004
CBM48	2.03 ± 0.25	1.40 ± 0.14	0.037
GH23	0.70 ± 0.11	2.55 ± 0.43	0.006

L group = low concentrate group; H group = high concentrate group. *p* < 0.01 indicates highly significant differences, 0.01 ≤ *p* < 0.05 indicates significant differences, and *p* ≥ 0.05 indicates insignificant differences.

#### 3.3.2 Go database functional annotation analysis

The results of the GO function of the rumen microbiome were shown in Supplementary Figure 1 and Supplementary Table 2. GO level 1 annotated results revealed that the relative abundance of MF in the H group was significantly higher than that in the L group (*p* = 0.025). In the present study, rumen microbial genes were annotated to 48 classes of GO level 2 function, the abundance of the top 20 functional classes was shown in Supplementary Table 2. Compared with the L group, the abundance of binding, organelle, biological regulation, response to stimulus, and signaling in the H group significantly increased (*p* < 0.05), and the abundance of macromolecular complex and membrane part significantly decreased (*p* < 0.05).

#### 3.3.3 KEGG database functional annotation analysis

Supplementary Figure 2 and Supplementary Table 3 showed the KEGG functions of the rumen microbiome. KEGG level 1 pathway analysis (Supplementary Figure 2) displayed that genetic information processing and organismal systems were significantly higher (*p* < 0.05), while metabolism and environmental information processing were significantly lower compared with the L group (*p* < 0.01). A total of 31 pathways of KEGG level 2 pathway analysis were annotated, and the relative abundance greater than 1% in each group was shown in Supplementary Table 3. Compared to the L group, the pathways of nucleotide metabolism, transport and catabolism, fold, sorting and degradation, translation, and endocrine system in the H group were significantly higher (*p* < 0.05), the overview, amino acid metabolism, carbohydrate metabolism, energy metabolism, cell motility, and membrane transport in the H group were significantly lower (*p* < 0.05).

#### 3.3.4 Differential expressed gene analysis of rumen microorganisms

In order to analyse the functional changes of goat rumen microbiome and their adaptive mechanisms to HCD, differential expressed genes (DEGs) statistics and DEGs functional enrichment analysis were performed on the rumen microbiome of two treatment groups. As shown in Supplementary Figure 3, the result showed that 424,100 DEGs were identified, of which 70,907 genes were up-regulated and 353,193 genes were down-regulated.

**Figure 7:**
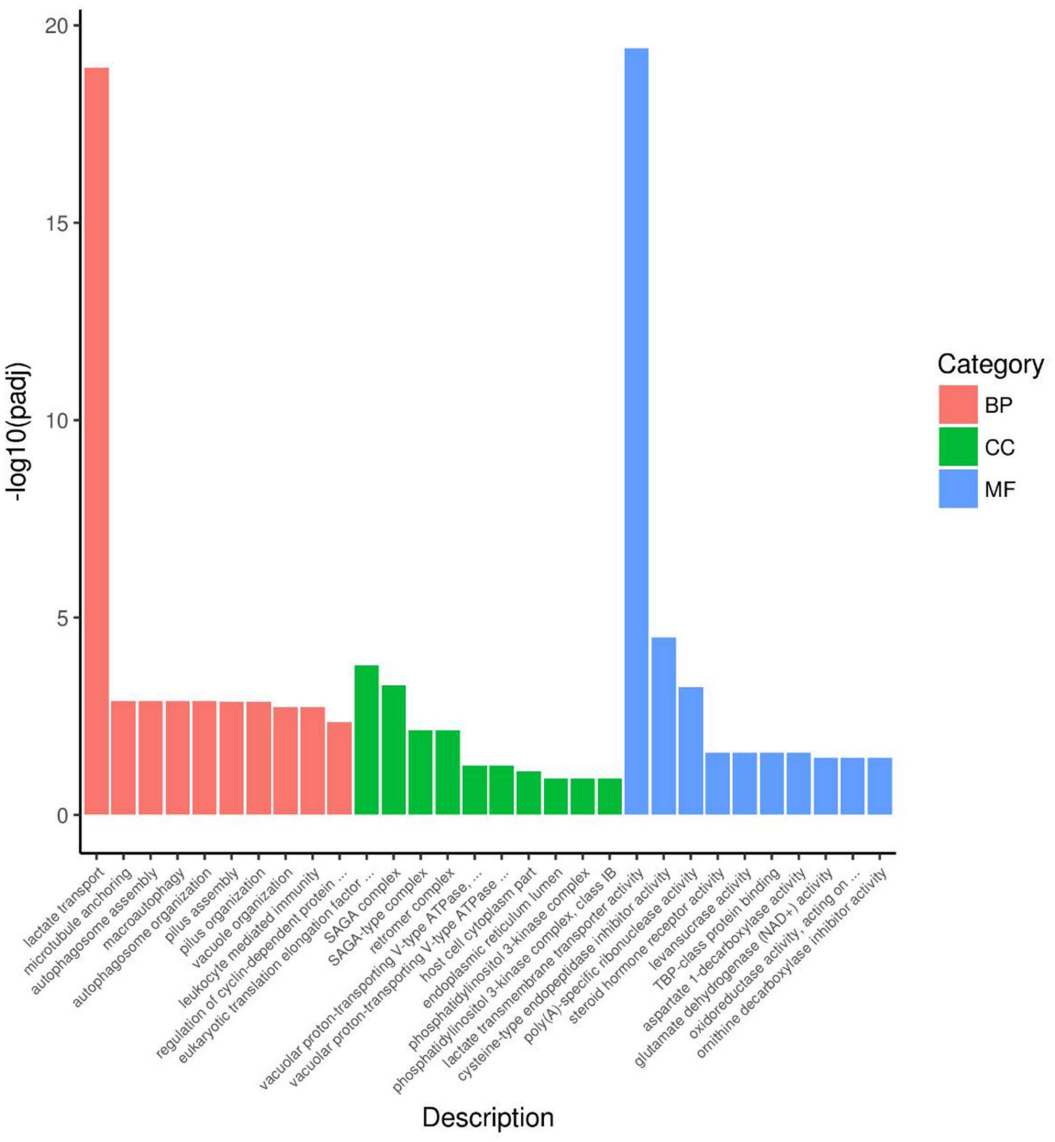
DGEs enrichment analysis of GO of rumen microbiome. BP = Biological process; CC = Cellular component; MF = Molecular function.

Figure 7 presented the top 10 functions of DEGs of GO, in which DEGs were mainly involved in lactate transport in the BP pathway, eukaryotic translation association factor 1 complex and SAGA complex in the CC pathway, and lactate transmembrane transport activity, cysteine-type endopeptidase inhibitor activity, and poly (A)-specific ribonuclease activity in the MF pathway.

**Figure 8:**
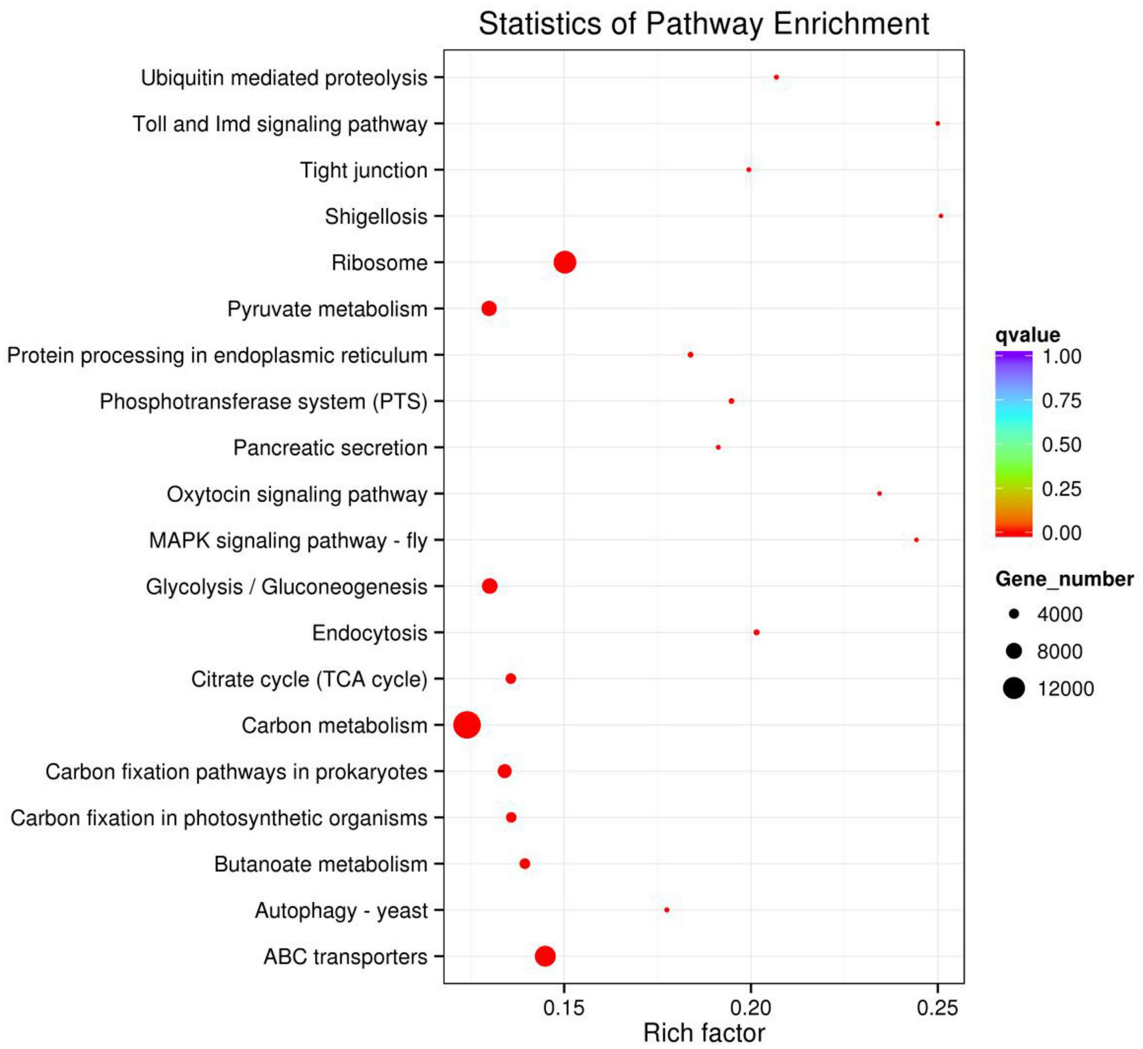
DEGs enrichment analysis of KEGG pathway of rumen microbiome.

The result of KEGG DEGs enrichment analysis showed that DEGs in rumen microbiome were mainly participated in metabolism pathways and genetic information processing pathways. Figure 8 displayed 20 pathways with significant enrichment. As shown in Figure 8, carbon metabolism (15,638 genes) enriched the most DEGs in rumen microbiome. In addition, ribosome (12,469 genes), and ABC transporters (11,249 genes) were also significant enrichment in rumen microbiome of two groups. According to the number of DEGs aligned to the KEGG pathway, there were 18 pathways with more than 1,000 DEGs (Supplementary Table 4). The result showed that DEGs were mainly enriched in carbon metabolism and energy metabolism.

## 4 Discussion

The rumen microbiome play an important role in the digestion of feed in ruminants (Castillo-González et al., 2014). Previous studies have shown that HCD significantly reduced rumen microbial diversity and alterd rumen microbial structure (Plaizier et al., 2017; Wang L. et al., 2020). As mentioned earlier, in this experiment, the alpha diversity of the H group was significantly reduced, and the beta diversity also differed significantly from the L group, indicating that feeding HCD to goats significantly altered the structure of their rumen microbiome. The results of the composition and intergroup differential analysis of rumen bacteria showed that the relative abundance of fiber-degrading bacteria in the H group, such as Firmicutes, Fibrobacteres (*Fibrobacter*, *Fibrobacter succinogenes*), *Bacteroides*, and *Ruminococcus flavefaciens*, was significantly lower than that in the L group, which was consistent with the study results of Thoetkiattikul et al. (2013), while the relative abundance of *Prevotella* and *Clostridium* increased. Among them, *Prevotella* is an important bacterium involved in the degradation of non-cellulosic substrates such as starch or proteins (Opdahl et al., 2018), and *Clostridium* actively participates in the digestion of starch, chitin, peptides, amino acids, and other substrates in the rumen (Pitta et al., 2014). These results may be due to the high starch content and low fiber content in HCD. The results of the composition and intergroup differential analysis of rumen fungi showed that the relative abundance of Basidiomycota, Neocallimastigomycota, *Mortierella*, *Mortierella* elongata, and *Gonapodya prolifera* was lower in the H group than in the L group. This may be because most of the fungi in the rumen are related to fiber degradation. Fungi have an extensive rhizoid system and can produce a variety of highly active enzymes, including cellulases, xylanases, and esterases, which can degrade and ingest plant cell walls through various physical and chemical methods (Han et al., 2019). Neocallimastigomycota, in particular, plays an important role in the degradation of fiber in ruminants, although its abundance in the rumen is low (Xue et al., 2022). All these results indicated that the reason for the changes in microbial structure and composition caused by HCD may be related to the nutrient differences in the feed, showing a “substrate-induced effect.” With the increase of concentrate in the diet, the relative abundance of fiber-degrading bacteria in the rumen decreased, while the relative abundance of starch-degrading bacteria increased, which form a digestive and metabolic structure beneficial for the host to utilize HCD.

**Figure 9:**
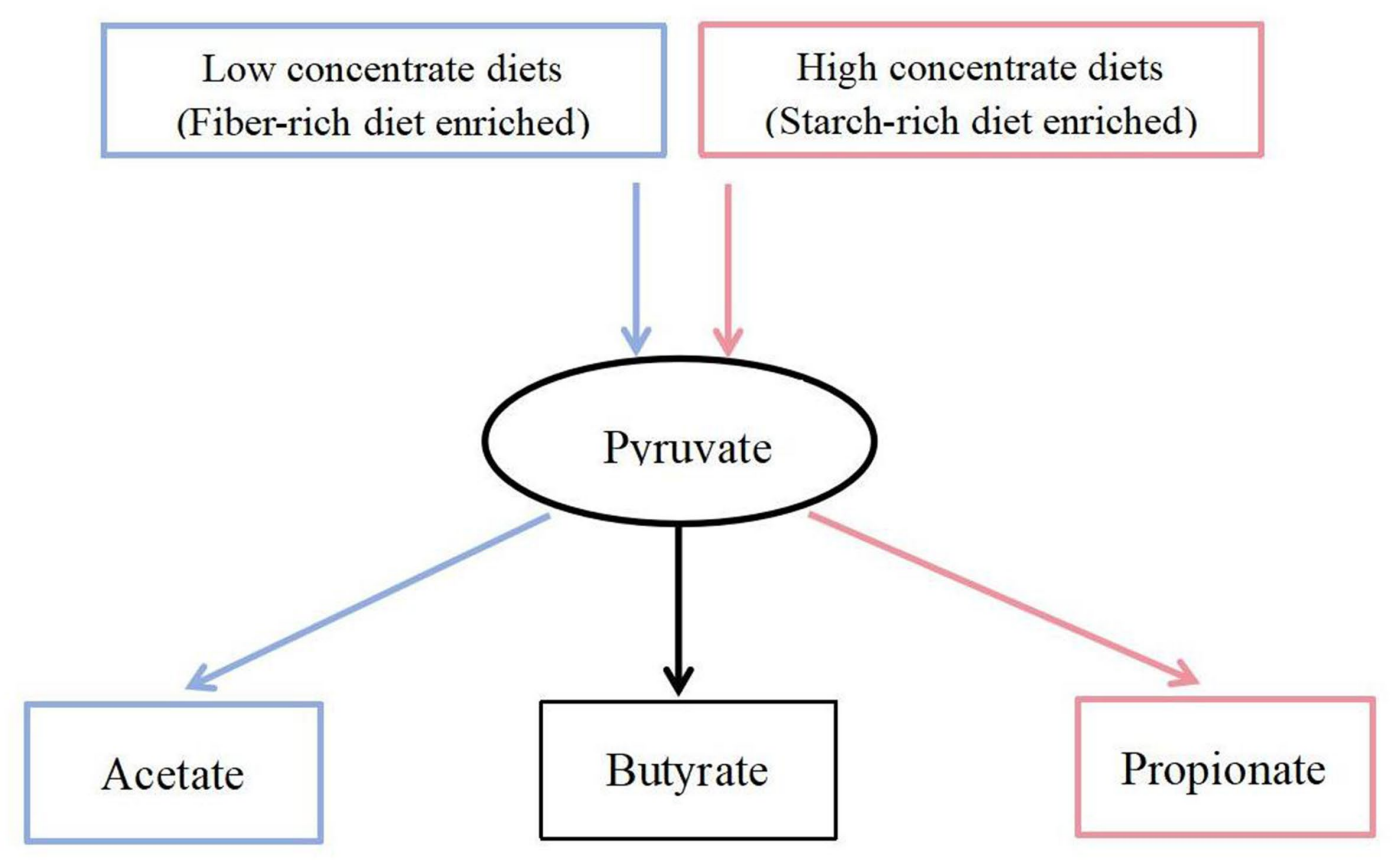
Carbohydrate fermentation. 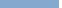 = Low concentrate diet enrichment pathway; 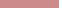 = High concentrate diet enrichment pathway; 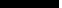 = Not enriched.

**Figure 10:**
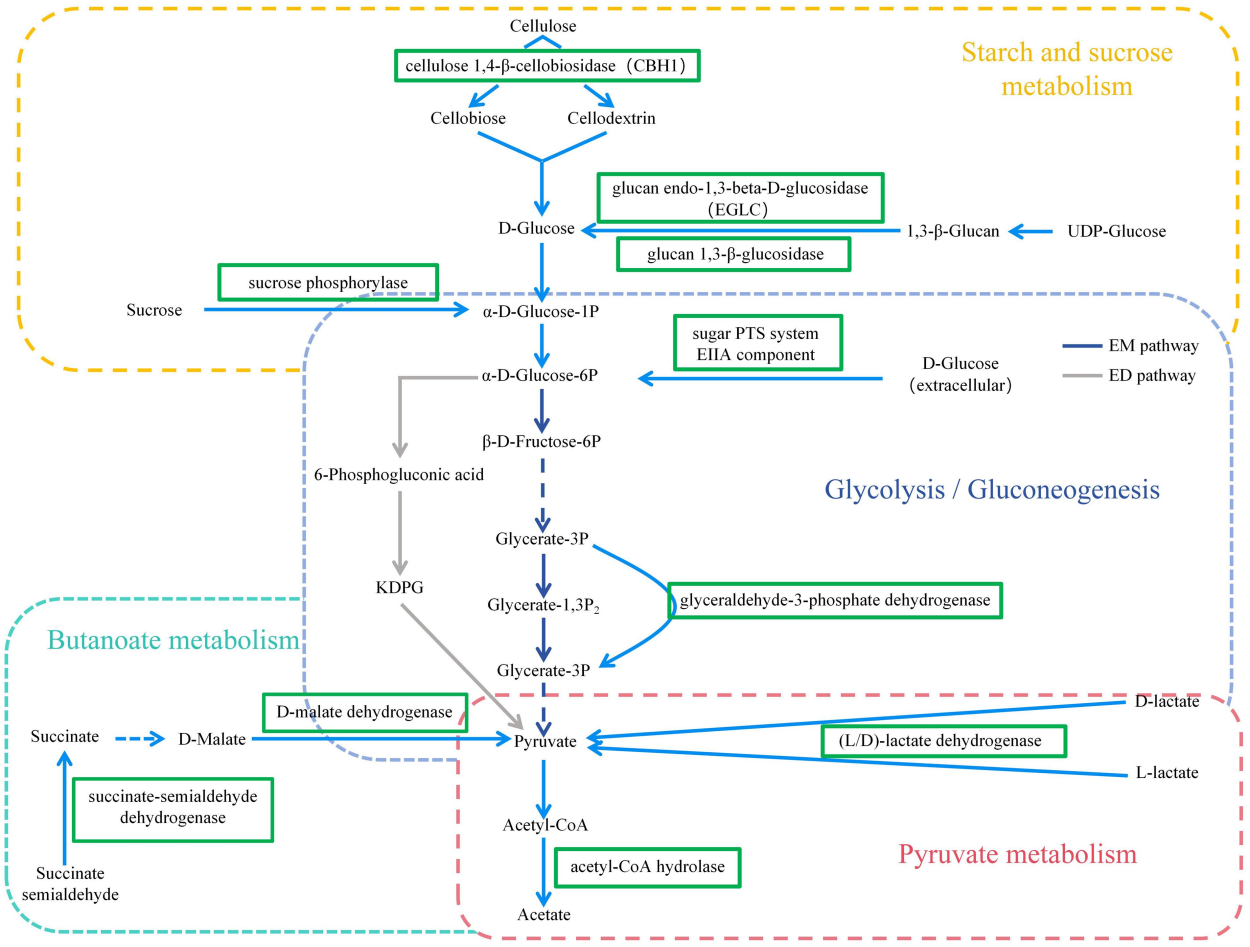
KEGG pathways of Carbohydrate metabolism (including Starch and sucrose metabolism, Glycolysis/Gluconeogenesis, Pyruvate metabolism, and Butanoate metabolism pathways). 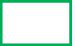 = down-regulated genes. EM pathway = Embedn-Meyerhof-Parnas pathway; ED pathway = Entner-Doudoroff pathway.

In this experiment, it was found that in the total VFAs composition of the rumen fluid, the proportion of acetate and the ratio of acetate to propionate were significantly reduced in the H group compared to the L group. Previous studies have also found that increasing the proportion of concentrate in the diet significantly reduced the acetate content and acetate to propionate ratio in the rumen (Hua et al., 2017; Zhang et al., 2017). Scholars have explained this result by stating that compared to concentrate, forage contained higher levels of cellulose and lower levels of starch. Cellulose fermentation mainly produced acetate, while starch fermentation mainly produced propionate. Therefore, when the proportion of concentrate in the diet was increased, the rumen fermentation pattern shifted from acetate-producing type to propionate-producing type (Zhang X. et al., 2020; Jiang et al., 2021). Based on a previous study (Li et al., 2022) we found that low- and high-concentrate diets produce different end products, but all of them produce different end products such as acetate, propionate, and butyrate by forming the intermediate pyruvate first, and then producing different end products through different pathways (Figure 9). However, the reason for this phenomenon is not clear. The results of this experiment showed that the reason why the dietary concentrate ratio affects the rumen fermentation pattern may be related to the changes in the expression levels of genes encoding carbohydrate metabolising enzymes secreted by rumen microbiome. Figure 10 provided a summary of the key nodes of carbohydrate degradation in this experiment and the enzymes whose gene expression levels had changed. In particular, the rumen microbiome of the H group showed a significant decrease in the gene expression level of acetyl-CoA hydrolase, which would directly reduce the production of acetate from acetyl-CoA. The gene expression levels of enzymes including glyceraldehyde-3-phosphate dehydrogenase in the glycolysis/gluconeogenesis pathway, (L/D)-lactate dehydrogenase in the pyruvate metabolism pathway, succinate-semialdehyde dehydrogenase and D-malate dehydrogenase in the butanoate metabolism pathway were also significantly reduced. These results directly or indirectly reduced the production of pyruvate, which was an important substrate for acetate production (Pan, 2012), and thus reduced the production of acetate. These outcomes may be related to changes in the composition and structure of rumen microbiome. This experiment found that the relative abundance of bacteria such as Fibrobacteres (*Fibrobacter*, *Fibrobacter succinogenes*), *Sarcina* (*Sarcina* sp. DSM 11001), and *Ruminococcus flavefaciens* in group H was significantly reduced. These bacteria were the main fiber-degrading bacteria in the rumen, all of which were able to break down cellulose to produce acetic acid. However, it was currently difficult to attribute the decrease in the gene expression level of a specific enzyme to the reduction in the quantity of a particular type of bacteria. Apart from VFAs, rumen microbiome also produced a large amount of lactate (LA) during the degradation of carbohydrates (Pitta et al., 2014; Opdahl et al., 2018). In this experiment, the LA level in the H group significantly increased, which was consistent with the results of Zhang et al. (2017). This may be due to the reduced gene expression of (L/D)-lactate dehydrogenase in the rumen microbial in the pyruvate metabolism pathway in group H (Figure 10), resulting in the inhibition of the pathway for the synthesis of pyruvate from lactate, which in turn resulted in the accumulation of LA. In addition, the enrichment analysis of DEGs in the GO database showed that there were significant differences in the expression of genes regulating lactate transport and lactate transmembrane transport activity by the rumen microbiome in the two groups, which may be another reason for the differences in rumen LA content between the groups in this experiment.

**Figure 11:**
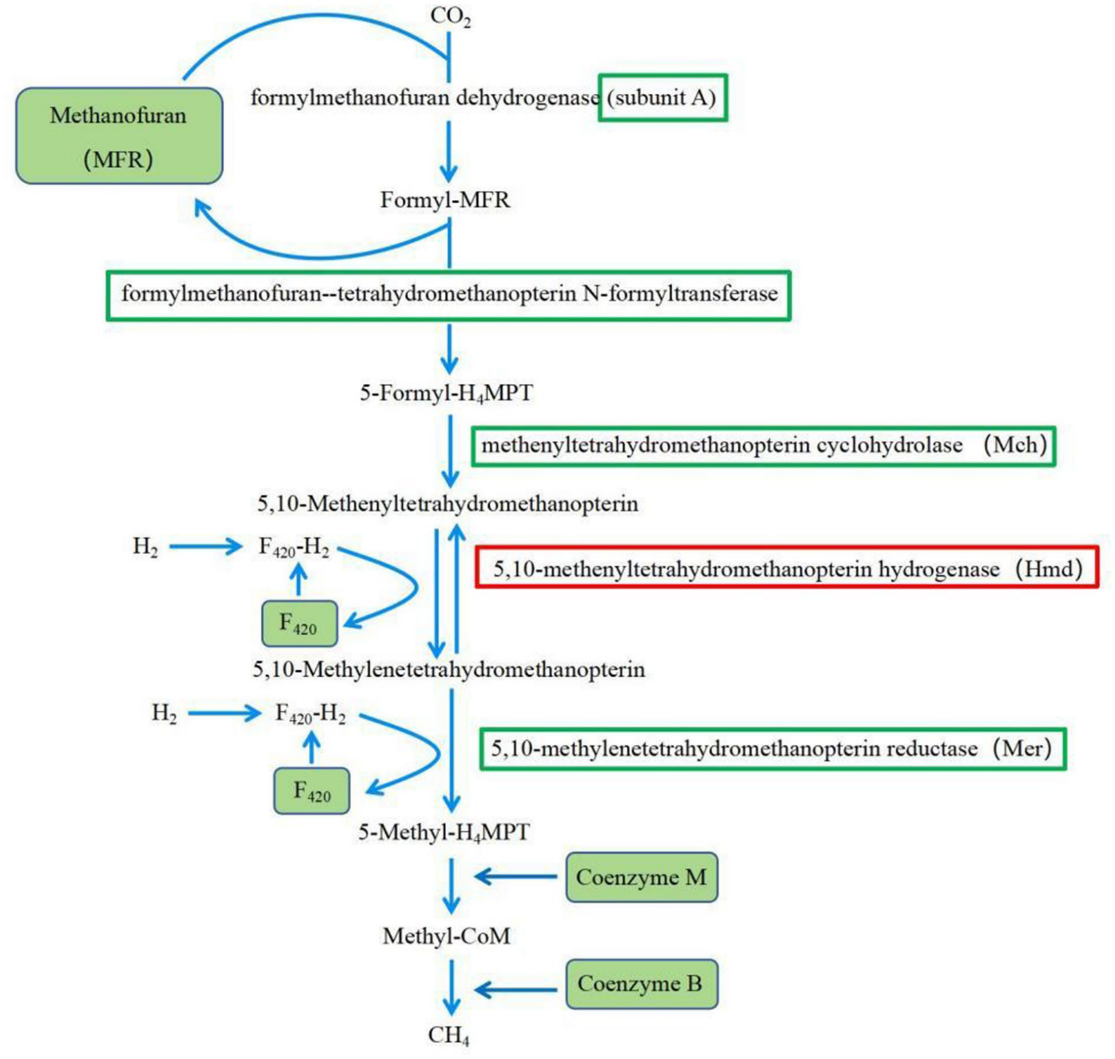
KEGG pathway of Methane metabolism. 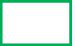 = down-regulated genes; 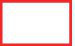 = up-regulated genes.

Methane (CH_4_) is one of the end products of ruminal fermentation, which has become a major research focus to reduce methane emissions in agriculture (Hristov et al., 2018; Ábrego-gacía et al., 2021). Previous studies have shown that when the proportion of concentrates in the diet exceeded 80%, only 3 to 4% of the total energy in the diet was converted to methane energy, while the proportion of total energy converted to methane energy was higher when forage was fed (Johnson and Johnson, 1995), which indicated that diet composition affected rumen CH_4_ production (Bannink et al., 2006; Beauchemin et al., 2008). The results of this experiment suggested that feeding HCD may reduce rumen CH_4_ production through down-regulation of key enzyme gene expression levels in the methane synthesis pathway (Figure 11). The gene expression levels of the enzymes involved in methane synthesis pathway of the H group, including the genes coding for formylmethanofuran-tetrahydromethanopterin N-formyltransferase, methenyltetrahydromethanopterin cyclohydrolase (Mch), and 5, 10-methylenetetrahydromethanopterin reductase (Mer), were significantly reduced. These enzymes are crucial for the reduction of CO_2_, and their reduced expression would inevitably lead to a decrease in the production of CH_4_ intermediate products, resulting in a lower CH_4_ yield. CoF_420_, CoB, and CoM are key coenzymes involved in the synthesis of CH_4_ by methanogenic archaea (Gao et al., 2016). In this experiment, the gene expression levels coding for these coenzymes were significantly reduced in the H group compared to the L group, which would also suppress CH_4_ generation. These results, for the first time, revealed the reasons for why feeding HCD to ruminants reduced rumen CH_4_ production from the perspective of gene expression.

Degradation of carbohydrates is the most important function of rumen microorganisms (Su et al., 2022). Carbohydrate-degrading enzymes produced by rumen microorganisms are collectively known as CAZymes (Bohra et al., 2019; Khatoon et al., 2022a). In this experiment, the predominant CAZymes family in the rumen was glycoside hydrolases (GHs, 43.84%), whose main function is to degrade glycosidic bonds in plant polysaccharides and lignocellulose (Manno et al., 2023). The functional annotation results at the family level in this experiment showed that the relative abundance of GHs genes in the L group was significantly higher than that in the H group. The unctional annotation results at the class level showed that the genes encoding GH94, GH32, GH43, GH13, and GH3 in group L were significantly higher than that in group H. GH94 contained cellobiose phosphorylase, laminaribiose phosphorylase, cellodextrin phosphorylase, and chitobiose phosphorylase, of which cellobiose phosphorylase accounts for about 75% (Isono et al., 2022). GH32, GH43, and GH3 are the major oligosaccharide—degrading enzymes, with GH32 annotated as β-furanofructosidase, and GH43 and GH3 annotated as endo-1,4-β-xylanase and α-L-arabinofuranosidase (Khatoon et al., 2022b; Zhong et al., 2023). All the above-mentioned enzymes are important in fiber degradation and played a crucial role in the degradation of cellulose and hemicellulose. Corresponding to the above results, the enrichment analysis of rumen microbial DEGs in this experiment showed that the expression of cellulose 1,4-β-cellobiosidase gene was significantly reduced in group H in the starch and sucrose metabolism pathway (Figure 10). The results of rumen microbial CAZymes annotation in this experiment were in agreement with the results of Yu (2021), whose experiment found that the abundance of fibre-degrading enzyme genes of rumen microbiome decreased and the abundance of oligosaccharides-degrading enzymes increased significantly with the increase in the proportion of dietary concentrates. The above results were also consistent with the results of the relative abundance of rumen microbiome, indicating that changes in the nutrient content of cellulose, starch, and other components in the diet will change the structure and composition of the rumen microorganisms, affecting the gene expression of rumen microorganisms, which will ultimately lead to changes in the function of the rumen microorganisms.

In addition, the results of this study showed that HCD improved production performance of goats, a result similar to Papi et al. (2011), who showed that lambs in the 30:70 (C70) group had the highest average daily weight gain, and that the feed conversion ratio was more effective in increasing the level of concentrates in the diet. This result may be due to the fact that HCD contains more energy to meet the production requirements of the animals. The rumen microbial structure changed with the diet structure, which favoured the utilisation of dietary nutrients by the host. And this study also found that feeding HCD down-regulated the expression level of key enzyme genes in the CH_4_ synthesis pathway and reduced CH_4_ synthesis, thus reducing the loss of total dietary energy converted to methane energy, which also favoured animal growth. Thus, feeding HCD may increase the efficiency of feed utilisation by the animal, thereby improving performance.

## 5 Conclusion

Feeding HCD significantly improved the production performance of goats. Feeding HCD altered the microbial structure and composition of the rumen, significantly affected the expression of genes involved in microbial carbohydrate metabolism processes, and may thus influence the type of rumen fermentation.

## Data availability statement

The names of the repository/repositories and accession number(s) can be found at: https://www.ncbi.nlm.nih.gov/, PRJNA1097788.

## Ethics statement

The animal study was approved by the Animal Policy and Welfare Committee of the Agricultural Research Organization of Sichuan Province, China. The study was conducted in accordance with the local legislation and institutional requirements.

## Author contributions

JM: Data curation, Writing – original draft, Writing – review & editing. LW: Writing – review & editing, Conceptualization, Funding acquisition, Methodology. ZW: Writing – review & editing, Investigation, Resources. BX: Writing – review & editing, Investigation, Resources. QP: Writing – review & editing, Investigation, Resources. RH: Investigation, Resources, Writing – review & editing. JX: Investigation, Resources, Writing – review & editing.

## Figures and Tables

**Table:** 

HCD	High concentrate diets
LA	Lactate
VFAs	Volatile fatty acids
L group	Low concentrate group
H group	High concentrate group
DM	Dry matter
CP	Crude protein
NDF	Neutral detergent fiber
ADF	Acid detergent fiber
EE	Ether extract
BW	Body weight
ADFI	Average daily feed intake
ADG	Average daily gain
F/G	Feed intake/gain
CAZymes	Carbohydrate-active enzymes
GO	Gene Ontology
KEGG	Kyoto Encyclopedia of Genes and Genomes
GHs	Glycoside hydrolases
GTs	Glycosyl transferases
PLs	Polysaccharide lyases
CEs	Carbohydrate esterases
AAs	Auxiliary activities
CBMs	Carbohydrate-binding modules
MF	Molecular function
CC	Cellular component
BP	Biological process
DEGs	Differential expressed genes

## Footnotes

^1^http://hmmer.janelia.org/search/hmmscan

^2^http://www.blast2go.com/b2ghome
