# Supplementary material for: High concentrate diets altered the structure and function of rumen microbiome in goats
Source: Front Microbiol. 2024 Jul 31;15:1416883. doi: 10.3389/fmicb.2024.1416883 (PMC11322510; doi:10.3389/fmicb.2024.1416883)
Supplement: Supplementary file 1 [file Data_Sheet_1.pdf]

Supplementary Material

1 Supplementary Figures and Tables

For more information on Supplementary Material and for details on the different file types accepted, please see here.

1.1 Supplementary Figures

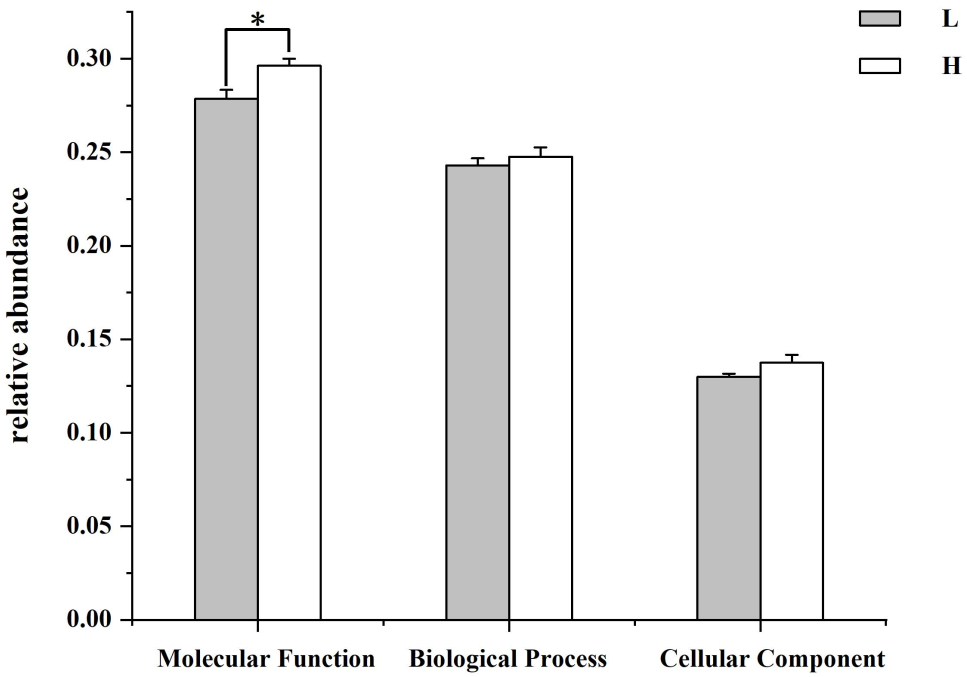

Supplementary Figure 1. Annotation result diagram of GO database of genes of rumen microbiome

L group = low concentrate group; H group = high concentrate group.

**Highly significant ($P < 0.01$); * Significant ($0.01 \leq P < 0.05$).

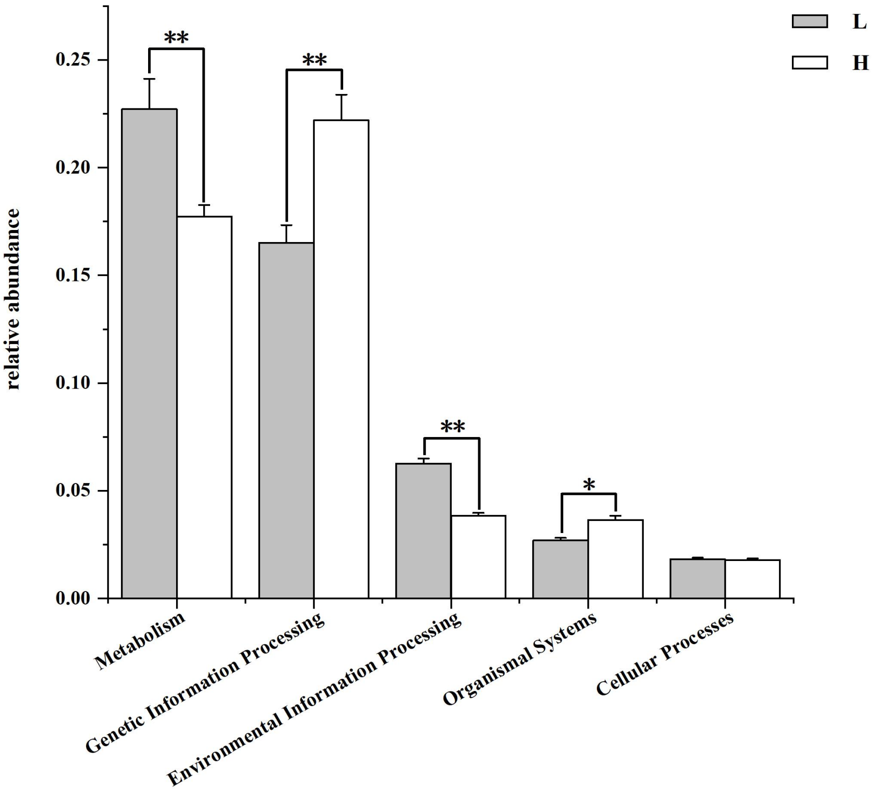

Supplementary Figure 2. Level 1 annotation results of rumen microbiome by KEGG database.

L group = low concentrate group; H group = high concentrate group.

**Highly significant (*P* < 0.01); * Significant (0.01 ≤ *P* < 0.05).

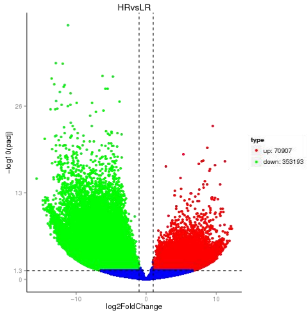

Supplementary Figure 3. Differential gene volcano diagram.

1.2 Supplementary Tables

Supplementary Table 1. The primer sequences of genes.

Gene name	Accession No.	Primer sequences (5′-3′)	Length(bp)
β-actin	NM_001314342.1	F: TCACGGAGCGTGGCTACAG<br>R: TTGATGTCACGGACGATTTCC	61
EIF4EBP1	NM_001285589.1	F: CCGGAGGTACCAGGATCATCT<br>R: CGTCTTGGTCACAGGTGAGTT	71
ACCS	XM_018059216.1	F: TGGCCAGGTTCCTGTCTTTCT<br>R: CCACCACATTCTCCGGTTTAAG	63
OXCT1	XM_013972785.2	F: CATTGCCAGTAAGCCGAGAGA<br>R:TGTGATTGCTTCCTCCAAGATAAAA	70
AACS	XM_005691340.3	F: CAGGTGTCTGGGCTCATGGT<br>R: ACGATTCCCCCGGTCTTG	58
APOA1	XM_018059749.1	F: GACCTTGGCTGTGCTCTTCCT<br>R: GGACGACTGTGGCTCATCTTG	73
APOC3	XM_005689483.3	F: CTACTCCTTCTTGCTGCCTTCCT<br>R: CCTCCTCGGCCTTGGTAGTT	64
APOA5	XM_018058993.1	F: CACGGAAAGGCTTCTGGGACT<br>R: CTCAAGTCTGTCTTTCAGGCTCG	110
HMGCR	XM_018053703.1	F:GCACGTCTACAGAAACTGCATATGA<br>R: TGGACTGAAAACGGATGTAAAGG	64

Supplementary Table 2. The level 2 annotation results of GO database of rumen microbiota genes.

Item		L group	H group	*P*-value
**Level 1**	**Level 2**			
Molecular Function	binding	9.74±0.07	10.67±0.22	0.010
	transporter activity	1.07±0.03	1.07±0.02	1.000
	catalytic activity	8.45±0.27	7.93±0.04	0.109
Cellular Component	cell part	5.19±0.10	5.51±0.15	0.078
	cell	5.40±0.10	5.72±0.15	0.078
	macromolecular complex	1.45±0.04	1.30±0.03	0.025
	organelle part	1.24±0.03	1.30±0.02	0.150
	membrane	2.15±0.03	2.16±0.02	1.000
	organelle	3.92±0.13	4.41±0.12	0.025
	membrane part	0.65±0.02	0.60±0.01	0.016
Biological Process	cellular process	10.74±0.07	10.93±0.12	0.262
	metabolic process	9.85±0.04	10.09±0.16	0.200
	regulation of biological process	2.29±0.03	2.41±0.04	0.055
	localization	2.64±0.14	2.45±0.04	0.337
	biological regulation	3.01±0.04	3.25±0.05	0.016
	cellular component organization or biogenesis	1.39±0.01	1.40±0.04	0.423
	response to stimulus	1.53±0.01	1.68±0.02	0.004

L group = low concentrate group; H group = high concentrate group.

$P < 0.01$ indicates highly significant differences, $0.01 \leq P < 0.05$ indicates significant differences, and $P \geq 0.05$ indicates insignificant differences.

Supplementary Table 3. The level 2 annotation results of KEGG database of rumen microbiota genes.

Item		L group	H group	*P*-value
**Level 1**	**Level 2**			
Metabolism	Overview	14.38±0.46	12.12±0.17	0.004
	Amino acid metabolism	5.27±0.17	4.49±0.17	0.010
	Biosynthesis of other secondary metabolites	1.48±0.04	1.39±0.05	0.150
	Carbohydrate metabolism	18.45±1.01	14.97±0.27	0.006
	Energy metabolism	13.29±0.51	10.50±0.13	0.004
	Lipid metabolism	1.28±0.04	1.25±0.03	0.749
	Metabolism of cofactors and vitamins	1.83±0.05	1.70±0.13	0.337
	Metabolism of other amino acids	1.28±0.04	1.25±0.03	0.423
	Nucleotide metabolism	3.56±0.09	3.94±0.08	0.010
Cellular Processes	Cell motility	1.05±0.10	0.41±0.06	0.004
	Transport and catabolism	0.79±0.08	1.40±0.12	0.006
Genetic Information Processing	Folding, sorting and degradation	3.32±0.07	4.57±0.21	0.004
	Translation	17.69±1.76	26.63±0.88	0.004
Organismal Systems	Endocrine system	2.36±0.12	3.06±0.10	0.006
Environmental Information Processing	Membrane transport	5.03±0.14	2.15±0.28	0.004

L group = low concentrate group; H group = high concentrate group.

$P < 0.01$ indicates highly significant differences, $0.01 \leq P < 0.05$ indicates significant differences, and $P \geq 0.05$ indicates insignificant differences.

Supplementary Table 4. DEGs enrichment analysis of KEGG pathway of rumen microbiome (The number of DEGs exceeds 1,000).

ID	Term	Type	DEGs	*P*-value	Corrected *P*-Value
ko01120	Microbial metabolism in diverse environments	Metabolism	22,593	5.03E-39	1.02E-37
ko01200	Carbon metabolism	Metabolism	15,638	9.44E-86	7.17E-84
ko03010	Ribosome	Genetic Information Processing	12,469	2.45E-286	7.44E-284
ko02010	ABC transporters	Environmental Information Processing	11,249	2.43E-212	3.69E-210
ko00500	Starch and sucrose metabolism	Metabolism	8,086	4.88E-31	6.46E-30
ko00010	Glycolysis / Gluconeogenesis	Metabolism	7,729	3.56E-68	1.54E-66
ko00620	Pyruvate metabolism	Metabolism	7,530	1.10E-65	3.70E-64
ko00720	Carbon fixation pathways in prokaryotes	Metabolism	6,659	3.07E-75	1.87E-73
ko00680	Methane metabolism	Metabolism	4,684	2.08E-31	2.88E-30
ko00650	Butanoate metabolism	Metabolism	4,405	4.28E-66	1.63E-64
ko00020	Citrate cycle (TCA cycle)	Metabolism	4,391	9.25E-55	2.56E-53
ko00710	Carbon fixation in photosynthetic organisms	Metabolism	4,361	1.07E-54	2.70E-53
ko00190	Oxidative phosphorylation	Metabolism	3,634	5.08E-21	4.98E-20
ko00051	Fructose and mannose metabolism	Metabolism	3,361	1.75E-19	1.57E-18
ko04144	Endocytosis	Cellular Processes	1,293	2.70E-88	2.73E-86
ko02060	Phosphotransferase system (PTS)	Environmental Information Processing	1,103	6.12E-69	3.10E-67

ko04141	Protein processing in endoplasmic reticulum	Genetic Information Processing	1,098	7.02E-58	2.13E-56
ko04066	HIF-1 signaling pathway	Environmental Information Processing	1,030	1.60E-25	1.87E-24
